# Intelligence prediction of integrated circuit reliability based on SSA-LSTM fusion architecture

**DOI:** 10.1371/journal.pone.0339394

**Published:** 2025-12-31

**Authors:** Ying Qian, Bing Liu, Beibei Su, Chunyan Zhang

**Affiliations:** Wuxi Vocational College of Science and Technology, Jiangsu Wuxi, China; Commonwealth Scientific and Industrial Research Organisation, AUSTRALIA

## Abstract

The relentless scaling of integrated circuits (ICs) into the nanoscale regime has intensified critical reliability challenges, such as Negative Bias Temperature Instability (NBTI), which manifests primarily as a progressive shift in the transistor threshold voltage ($\Delta V_{th}$). This study focuses on the prognostics of digital integrated circuits (ICs) where Metal-Oxide-Semiconductor Field-Effect Transistors (MOSFETs) serve as the fundamental building blocks. Accurate prognostics of device degradation are paramount for predicting circuit lifetime, yet traditional models struggle with the nonlinearity of the degradation process. To address this challenge, a novel fusion architecture is proposed that synergistically combines the Sparrow Search Algorithm (SSA) with a Long Short-Term Memory (LSTM) network. The resulting SSA-LSTM model automates the optimization of crucial LSTM hyperparameters—including the number of hidden units, learning rate, and iteration count—thereby enhancing the capability to learn complex temporal degradation patterns. Empirical Mode Decomposition (EMD) is further integrated as a pre-processing step to denoise the temporal data. This model significantly reduces the average absolute error of $\Delta V_{th}$ prediction, outperforming benchmark models such as PSO and EMD-PSO. It can accurately capture the nonlinear trajectory of degradation curves and has high sensitivity for early anomaly detection, providing a high-precision data-driven solution for the reliability evaluation and prediction of digital ICs.

## 1 Introduction

Integrated Circuits (ICs) serve as the fundamental building blocks of the modern digital economy, underpinning advancements in computing, communications, and consumer electronics through their unparalleled capabilities in performance, miniaturization, and efficiency [1–3]. However, as IC feature sizes shrink to the nanoscale, their operational reliability is increasingly threatened by physical degradation mechanisms. Phenomena such as Negative Bias Temperature Instability (NBTI), Hot Carrier Injection (HCI), and Time-Dependent Dielectric Breakdown (TDDB) induce gradual and often irreversible performance shifts, with the threshold voltage drift ($\Delta V_{th}$) of transistors being a primary and critical manifestation [4–6]. The repercussions are severe; for instance, $\Delta V_{th}$ drift can lead to timing violations, increased power consumption, and ultimately, functional failure, accounting for a significant portion of field returns in demanding sectors like automotive electronics.

The reliability assessment of ICs at the nanoscale is severely hampered by three fundamental limitations. First, established physical models become inadequate, failing to capture the complex interplay of multiple stress factors. Furthermore, conventional test methods, such as production line burn-in (PLBI), grow prohibitively expensive and inefficient for complex AI Systems-on-Chip (SoCs). Compounding these issues, shallow machine learning techniques lack the capacity to decode the intricate spatiotemporal patterns inherent in heterogeneous reliability data. Among various monitored parameters, the threshold voltage shift ($\Delta V_{th}$) has emerged as a quintessential metric. Its prominence stems from its direct correlation to dominant failure mechanisms like NBTI and HCI [7], its profound impact on circuit performance (governing switching speed and power consumption), and its practical measurability throughout the device lifecycle using standard automated test equipment (ATE) [8,9].

In this context, data-driven methods, particularly deep learning, offer a viable alternative for automatically learning degradation dynamics from massive datasets without requiring explicit physical equations [10–12]. While various data-driven approaches exist for $\Delta V_{th}$ prediction—including physics-informed statistical models like Bayesian updating and Gaussian Process Regression [13,14] and shallow machine learning methods [15,16]—these often fail to fully capture the complex degradation trajectory. Long Short-Term Memory (LSTM) networks are particularly suitable for modeling such temporal sequences due to their inherent architecture. However, their predictive performance remains highly sensitive to hyperparameter configuration [17–19], and manual tuning typically yields suboptimal results while being labor-intensive and dependent on expert intuition.

Recent research highlights the efficacy of hybrid models that integrate optimization algorithms with deep learning. For instance, Hang et al. [20] successfully combined Variational Mode Decomposition (VMD), SSA, and LSTM for safety prediction in aerial refueling, demonstrating the power of signal processing and intelligent optimization. Similarly, Ma et al. [21] developed a Mechanics-Guided Data-Driven model that integrates physical mechanisms with deep learning for high-accuracy prediction of complex geophysical processes. Inspired by these advancements, this study utilizes the Sparrow Search Algorithm (SSA) [22–24] to automatically and adaptively optimize the hyperparameters of the LSTM network. This fusion mitigates the guesswork in model configuration and enhances prediction robustness.

According to the above literature investigation, the method proposed in this paper is effective in predicting transistor threshold voltage drift, and the principal innovations of this work are threefold:

1. **Novel SSA-LSTM Fusion Architecture with Automated Hyperparameter Optimization**: Introduces an intelligent framework that synergistically combines the global search capability of the Sparrow Search Algorithm with the superior temporal modeling of Long Short-Term Memory networks, enabling automated optimization of critical hyperparameters for robust IC reliability prediction.
2. **Precise $\Delta V_{th}$-Centered Degradation Tracking with Physical Foundation**: Establishes the threshold voltage shift as a direct and physically-grounded core metric, providing a critical linkage between transistor-level aging phenomena and circuit-level performance degradation through integrated mathematical modeling of power-law kinetics and acceleration effects.
3. **Demonstrated Empirical Superiority with High Prognostic Sensitivity**: Validates significant performance advantages through rigorous comparative experiments, showing substantial prediction error reduction on accelerated aging data while maintaining exceptional sensitivity for early anomaly detection in practical intelligent health management systems.

The paper is organized as follows: Sect 2 develops the mathematical foundation for threshold voltage drift modeling, establishing both power-law and acceleration models. Sect 3 details the proposed SSA-LSTM fusion architecture, encompassing data preprocessing techniques, algorithmic fundamentals, and model integration strategies. Sect 4 presents the experimental setup and provides a comprehensive performance analysis through comparative validation. Finally, Sect 5 concludes the work by highlighting key findings and their implications for IC reliability prognostics.

## 2 Mathematical modeling establishment

### 2.1 Physics informed degradation modeling

The core of our prognostic approach is not purely data-driven but is informed by well-established physical models of IC degradation. This ensures that the LSTM network learns representations that are physically plausible.

The temporal evolution of the threshold voltage shift, $\Delta V_{th}$, is predominantly described by a power-law model, which is empirically well-established for capturing degradation mechanisms like NBTI [25]:

$$\Delta V_{th}(t)=A\cdot t^{n}$$ (1)

where *t* is the stress time, *A* is a pre-factor or proportionality constant, and *n* is a dimensionless empirical exponent (fitting parameter).

**Remark 1:** All experiments are conducted under controlled stress conditions, where temperature and gate voltage are kept constant. This avoids the need for multi-variable modeling, which would significantly increase the complexity of the analysis.

**Remark 2:** Samples collected under different stress conditions are assumed to be independent and comparable. It is further assumed that environmental factors such as humidity, electromagnetic interference, and equipment deviations have been minimized to avoid introducing systematic errors in the measurement of $\Delta V_{th}$.

### 2.2 Accelerated model of life prediction

The parameter *A* in the power-law model (1) is not a true constant but a stress-dependent prefactor. Its temperature dependence is classically described by the Arrhenius relation. In this context, *A*(*T*) represents the value of the prefactor at a specific temperature *T*, and is modeled as:

$$A(T)=A_{0}(V)\cdot \exp\left(-\frac{E_{a}}{k_{B}T}\right)$$ (2)

where $A_{0}(V)$ is a voltage-dependent coefficient, *E*_*a*_ is the activation energy (e.g., 0.1-0.2 eV for interface trap generation and 0.8-1.0 eV for hole trapping in NBTI [26]), *k*_*B*_ is Boltzmann’s constant, and *T* is the absolute temperature.

Similarly, the voltage dependence of the prefactor is frequently described by an exponential model, where the stress voltage *V* is the gate-to-source voltage $V_{gs}$ applied during the accelerated test [27]:

$$A(V_{gs})\propto \exp(\gamma V_{gs})$$ (3)

Here, γ is the voltage acceleration factor, which typically ranges from 1 to 10 ${\text{V}}^{-1}$ depending on the technology and oxide thickness [28].

Integration with Data-Driven Approach: Our SSA-LSTM model does not attempt to directly fit these equations. Instead, it learns the complex, nonlinear functional ℱ that maps a sequence of past $\Delta V_{th}$ measurements and the corresponding stress conditions (*T*, $V_{gs}$) to future values:

$$\left[\Delta V_{th}(t+1),\ldots ,\Delta V_{th}(t+k)]=F\text{LSTM}([\Delta V_{th}(t-p),\ldots ,\Delta V_{th}(t)];T,V_{gs};\Theta \right)$$ (4)

where Θ are the model parameters optimized by SSA. By training on data from multiple stress conditions, the model inherently learns to generalize the effects encapsulated in the physical acceleration models, enabling predictions under varying operational profiles.

**Assumption 1:** The failure threshold for chip lifetime prediction is predefined and well-established. It is assumed that the end of the chip’s useful life occurs when the threshold voltage shift $\Delta V_{th}$ exceeds a specified limit (e.g., 50 mV), which should be determined based on reliable reliability standards or manufacturing specifications.

**Assumption 2:** The trained LSTM model is assumed to effectively capture the nonlinear temporal evolution of $\Delta V_{th}$ and provide accurate short-term predictions. Furthermore, the LSTM outputs are assumed to be compatible with power-law model fitting, enabling integration with acceleration models for lifetime extrapolation. The LSTM hyperparameters optimized by the SSA are presumed to offer near-optimal global solutions with good generalization performance on unseen data.

## 3 Threshold voltage drift $\Delta V_{th}$ prediction model

### 3.1 Forecasting target

**Fig 1:**
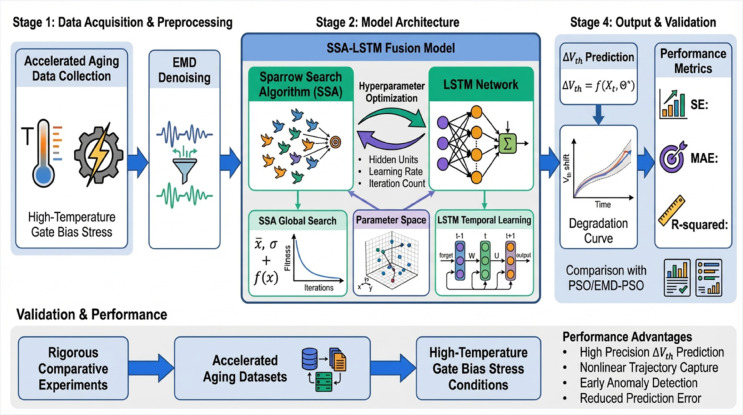
SSA-LSTM IC reliability prognostics architecture.

The prediction objective of this study is to perform high-precision modeling and forecasting of the threshold $\Delta V_{th}$ in transistors within integrated circuits, which serves as a key indicator of device aging and performance degradation. By constructing a LSTM prediction model optimized via the SSA, referred to as SSA-LSTM, the goal is to dynamically predict the nonlinear degradation process of $\Delta V_{th}$. This approach aims to provide accurate support for intelligent reliability prognostics and health management of integrated circuits. The prediction logic of this study is illustrated in Fig 1.

### 3.2 Data preprocessing

To enhance signal quality and extract meaningful degradation patterns from noisy voltage shift data, this study investigates two signal decomposition techniques: VMD [29] and EMD [30]. Both are widely used for nonstationary and nonlinear signal analysis, offering effective time-frequency decomposition capabilities.

#### 3.2.1 Variational mode decomposition.

VMD is a non-recursive, variational technique that decomposes a signal into a finite set of band-limited intrinsic mode functions (IMFs), each centered around an adaptive center frequency. Unlike EMD, VMD is defined as a constrained optimization problem.

The decomposition is formulated as a constrained variational problem:

$$\min_{\left\{u_{k}\right\},\left\{\omega_{k}\right\}}\left\{\sum_{k}{\left\|\partial_{t}\left[\left(\delta (t)+\frac{j}{\pi t}\right)*u_{k}(t)\right]e^{-j\omega_{k}t}\right\|}_{2}^{2}\right\}\;\text{ s.t. }\;\sum_{k}u_{k}=f$$ (5)

where the symbol $|\cdot {|}_{2}$ denotes the *L*_2_ norm of a signal, and $|\cdot {|}_{2}^{2}$ represents its square, which corresponds to the signal’s energy. *u*_*k*_(*t*) represents the *k*-th mode function to be extracted from the original signal *f*(*t*), while $\omega_{k}$ denotes its corresponding center frequency. The decomposition utilizes the Dirac delta function δ(t) and the convolution operator * to shift the signal into the frequency domain. The imaginary unit *j* and the partial time derivative $\partial_{t}$ are used in constructing the analytic signal for each mode, enabling the algorithm to isolate modes with minimal spectral overlap.

**Remark 3:** VMD ensures that each mode is compact in the frequency domain, which enhances separation accuracy. However, its effectiveness depends on carefully selecting the number of decomposition modes and the penalty parameter, which may require empirical tuning.

#### 3.2.2 Empirical mode decomposition.

EMD is an adaptive, data-driven approach that decomposes a signal into a set of intrinsic mode functions (IMFs) without requiring a predefined basis or model. It is particularly suited for nonlinear and nonstationary time series.

The decomposition process follows a “sifting" algorithm:

1. Identify all local extrema (maxima and minima).

2. Construct upper and lower envelopes using cubic spline interpolation.

3. Compute the mean envelope:

$$m(t)=\frac{e_{\max}(t)+e_{\min}(t)}{2}$$ (6)

4. Extract the detail:

h(t)=f(t)−m(t) (7)

5. Check whether *h*(*t*) satisfies IMF conditions. If not, repeat steps 1-4 on *h*(*t*).

6. Subtract the IMF from the signal and repeat for the residual.

The final decomposition yields:

$$f(t)=\sum_{i=1}^{n}{\mathrm{IMF}}_{i}(t)+r_{n}(t)$$ (8)

where ${IMF}_{i}(t)$ refers to the *i*-th Intrinsic Mode Function, which represents a simple oscillatory component extracted from the original signal. These IMFs capture localized time-frequency information at different scales. The term *r*_*n*_(*t*) denotes the final residual signal after extracting *n* IMFs, typically representing the long-term trend or non-oscillatory baseline of the original data. Together, the IMFs and the residual form a complete and adaptive decomposition of the input signal.

**Remark 4:** EMD is particularly advantageous for analyzing nonlinear and nonstationary signals, such as degradation curves in electronic components, due to its fully data-driven and adaptive nature. Its high temporal resolution allows precise tracking of time-varying signal characteristics, while its robustness to variations in mode shapes ensures reliable extraction of degradation trends. These properties make EMD highly suitable for preprocessing tasks in reliability prediction models, where capturing subtle changes in early-stage degradation is critical.

### 3.3 Basic principle of LSTM

In the field of deep learning research on Q tasks, Recurrent Neural Networks (RNNs) are widely utilized due to their powerful capabilities in handling sequential data, such as in speech recognition and natural language processing [31–33]. However, traditional RNNs often suffer from the vanishing gradient problem when dealing with long sequences, making it difficult to learn long-range dependencies. To address this challenge, LSTM networks were introduced as an enhanced RNN architecture. With their gate-based mechanism, LSTMs effectively mitigate this issue and have become a key technology in sequence modeling.

#### 3.3.1 LSTM gating mechanism.

**Fig 2:**
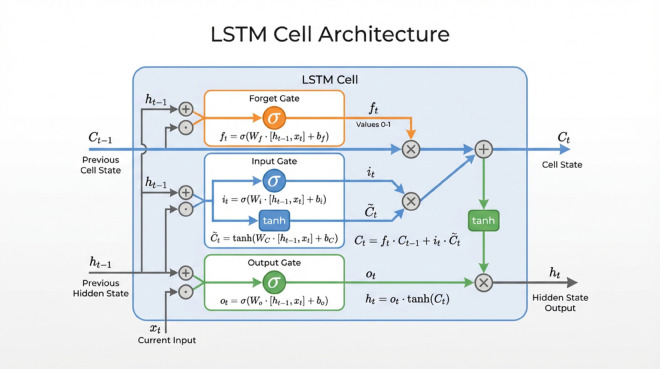
The LSTM logical structure diagram.

The LSTM network addresses the short-term memory and gradient vanishing problems of traditional RNNs through its gating mechanism, comprising the forget, input, and output gates. These gates collectively regulate the retention, update, and output of information, enabling the network to effectively capture long-term dependencies. The logical structure of LSTM is depicted in Fig 2.

**(1) Forgetting gate**

The forget gate controls which information from the long-term cell state should be retained or discarded. It takes the current input and the previous hidden state as inputs, passing them through a Sigmoid function to generate values between 0 and 1. Values close to 0 imply that the corresponding information in the cell state will likely be discarded, while values close to 1 indicate that the information will be retained. The calculation formula of forgetting gate is as follows:

$$f_{t}=\sigma \left(W_{f}\cdot \left[h_{t-1},x_{t}\right]+b_{f}\right)$$ (9)

where *W*_*f*_ and *b*_*f*_ are the weight matrix and bias vector of forgetting gate, respectively.

**(2) Input gate**

The input gate determines which parts of the current input information should be incorporated into the cell state. It processes the current input along with the previous hidden state through a Sigmoid function to produce the update proportions, and simultaneously uses a Tanh function to preprocess the input. Multiplying these two results yields the information that will actually be updated into the cell state.

$$i_{t}=\sigma \left(W_{i}\cdot \left[h_{t-1},x_{t}\right]+b_{i}\right)$$ (10)

$${\tilde{C}}_{t}=\tanh\left(W_{C}\cdot \left[h_{t-1},x_{t}\right]+b_{C}\right)$$ (11)

where $W_{i},b_{i},W_{C}$ and *b*_*C*_ are the correlation weight matrix and bias vector of the input gate respectively.

According to the results of forgetting gate and input gate, the cell state is updated. The specific formula is as follows:

$$C_{t}=f_{t}⊙C_{t-1}+i_{t}⊙{\tilde{C}}_{t}$$ (12)

where ⊙ stands for element-by-element multiplication.

**(3) Output gate**

The output gate controls which parts of the cell state will be outputted as the current hidden state. It processes the current input and previous hidden state through a Sigmoid function to determine the proportion of information to be outputted. This proportion is then multiplied by the cell state activated through a Tanh function to obtain the final hidden state at the current timestep.

$$o_{t}=\sigma \left(W_{o}\cdot \left[h_{t-1},x_{t}\right]+b_{o}\right)$$ (13)

$$h_{t}=o_{t}⊙\tanh\left(C_{t}\right)$$ (14)

where *W*_*o*_ and *b*_*o*_ are the weight matrix and bias vector of the output gate, respectively.

### 3.4 Sparrow search algorithm

**Fig 3:**
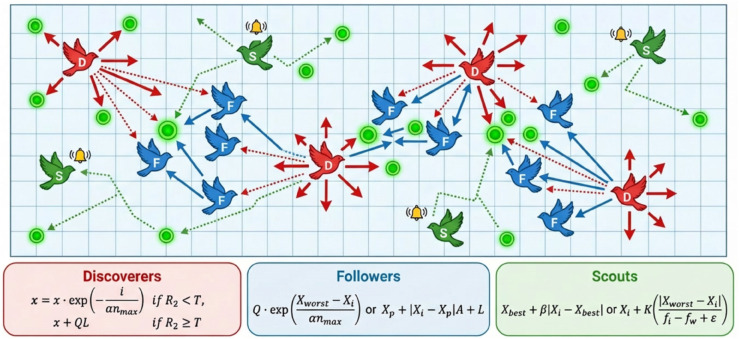
The schematic diagram of sparrow search algorithm.

The SSA is a swarm intelligence optimization technique that mimics sparrow foraging and anti-predation behavior. It employs a population divided into explorers (searching for food), followers (exploiting found resources), and vigilants (issuing random warnings). This structure ensures effective exploration-exploitation balance, allowing SSA to efficiently solve complex optimization problems [20,23,34], as shown in Fig 3.

#### 3.4.1 Description of mathematical formula of SSA.

During the foraging process, discoverers exhibit a broader search capability, actively guiding followers and continuously updating their own positions based on memory, thereby improving the efficiency of locating food sources. The position update follows the iterative formula below.

$$x_{i,d}^{\left(t+1\right)}=\left\{ \begin{array}{cc} x_{i,d}^{\left(t\right)}\cdot \exp\left(\frac{-i}{\alpha n_{\max}}\right), & R_{2}<T\\ x_{i,d}^{\left(t\right)}+QU, & R_{2}⩾T \end{array}\right.$$ (15)

where *Q* is a random number drawn from a standard normal distribution, which introduces a scalar perturbation to the position. $x_{i,d}^{\left(t\right)}$ denotes the *d*-th dimensional position of the *i*-th sparrow in the *t*-th generation of the population, where d=1,2,3,…,D and *D* is the total number of dimensions. α is a uniformly distributed random number in the interval [0,1]. $n_{\text{max}}$ is the maximum number of iterations, *R*_2_ is an alertness factor drawn from a uniform distribution over [0,1], *T* represents the alert threshold, typically ranging from 0.5 to 1, and *U* is a 1×D matrix with all elements equal to 1.

During the foraging process, followers continuously monitor the behavior of discoverers. Once a discoverer locates a new food source, the followers immediately move toward the updated position to compete for the resource. The position update rule for followers is given by Eq (16).

$$x_{i,d}^{\left(t+1\right)}=\left\{ \begin{array}{c} Q\cdot \exp\left(\frac{X_{\text{worst }}^{\left(t\right)}-X_{i,d}^{\left(t\right)}}{\alpha n_{\max}}\right),i>\frac{n}{2}\\ X_{p}^{\left(t+1\right)}+\left|X_{i,d}^{\left(t\right)}-X_{p}^{\left(t+1\right)}\right|A^{+}U,i⩽\frac{n}{2} \end{array}\right.$$ (16)

where *X*_*p*_ represents the best position currently occupied by a discoverer, while $X_{\text{wors}}$ denotes the worst position in the population. *A* is a 1×D matrix with each element randomly selected from the interval (–1,1), and $A^{+}=A^{T}(AA^{T}){\;}^{-1}$ denotes the generalized inverse of matrix *A*. The parameter *n* refers to the total number of sparrows in the population.

Considering the potential threat of predators during foraging, approximately 10% to 20% of the sparrows are designated as scouts. These scouts are initially distributed randomly, and if danger is detected, they will immediately abandon the current food source and relocate to a safer position. The position update rule for scouts is defined by Eq (17).

$$x_{i,d}^{\left(t+1\right)}=\left\{ \begin{array}{c} X_{\text{best }}^{\left(t\right)}+\beta \left|X_{i,d}^{\left(t\right)}-X_{\text{best }}^{\left(t\right)}\right|,f_{i}>f_{g}\\ X_{i,d}^{\left(t\right)}+K\left(\frac{\left|X_{\text{worst }}^{\left(t\right)}-X_{i,d}^{\left(t\right)}\right|}{f_{i}-f_{w}+\varepsilon }\right),f_{i}=f_{g} \end{array}\right.$$ (17)

where $X_{\text{best}}$ denotes the current global best position found within the population. $f_{g}$ and $f_{w}$ represent the fitness values of the globally best and worst individuals, respectively, while *f*_*i*_ is the fitness of the *i*-th sparrow. β is a random variable that follows a standard normal distribution, and *K* is a randomly selected value from the interval [–1,1] that determines the movement direction of the sparrow. ε is a small constant introduced to prevent division by zero.

### 3.5 SSA-LSTM prediction model

**Fig 4:**
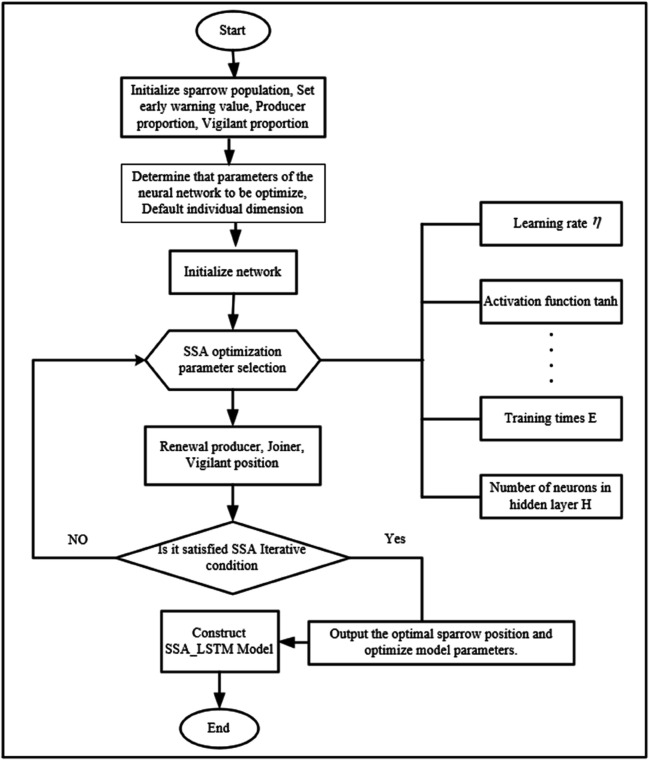
The process of SSA-LSTM prediction model.

After applying the data preprocessing method described in Sect 3.2, the dataset is divided into training and testing sets, which are then loaded into the corresponding registers. The SSA is subsequently employed to optimize the key hyperparameters of the LSTN model. Once the optimal hyperparameters are obtained, they are fed into the LSTM model to construct predictive models for different output targets. The overall workflow of the SSA-LSTM prediction model is illustrated in Fig 4.

### 3.6 SSA-LSTM hyperparameter optimization strategy

The optimization of LSTM hyperparameters using SSA was formulated as a constrained search problem to minimize the prediction error on the validation set.

#### 3.6.1 Search space: The SSA was configured to optimize three critical hyperparameters.

- Number of hidden units: Discrete search space [32, 64, 128, 256].

- Initial Learning Rate: Continuous log-space [1e-4, 1e-1].

- L2 Regularization Factor: Continuous log-space [1e-5, 1e-2].

### 3.7 SSA configuration and execution

The sparrow population size was set to 30, and the algorithm was run for 50 iterations. The proportion of discoverers was set to 20% and the scouts to 10% of the population. The algorithm was terminated when the fitness improvement fell below a tolerance of $1\;\times \;10^{-6}$ for 10 consecutive iterations. The best-performing parameter set from this automated search was then used to train the final LSTM model on the combined training and validation sets before evaluation on the test set.

### 3.8 Predictive performance evaluation index

In the fields of machine learning and data analysis, several statistical metrics are commonly used to evaluate the predictive performance of a model. The following are three widely used and essential evaluation metrics:

**1. Root Mean Square Error (RMSE)**

RMSE measures the magnitude of the difference between predicted and actual values. A smaller RMSE indicates that the model’s predictions are closer to the true values, implying better performance. It is calculated as:

$$RMSE=\sqrt{\frac{1}{n}\sum_{i=1}^{n}{\left(x_{p,i}-x_{r,i}\right)}^{2}}$$ (18)

where $x_{p,i}$ is the predicted value, $x_{r,i}$ is the actual value, and *n* is the total number of data points.

**2. Mean Absolute Percentage Error (MAPE)**

MAPE expresses the average prediction error as a percentage of the actual values, providing an intuitive understanding of how large the errors are relative to the true values. A lower MAPE indicates higher overall prediction accuracy. The formula is:

$$MAPE=\frac{1}{n}\sum_{i=1}^{n}\left|\frac{x_{p,i}-x_{r,i}}{x_{r,i}}\right|\times 100\%$$ (19)

**3. Coefficient of Determination (*R***^**2**^)

*R*^2^, also known as the coefficient of determination, evaluates how well the model explains the variance in the observed data. An *R*^2^ value closer to 1 indicates a better fit, whereas a value of 0 means the model fails to capture any variability. The formula is:

$$R^{2}=1-\frac{\sum_{i=1}^{n}{\left(x_{p,i}-x_{r,i}\right)}^{2}}{\sum_{i=1}^{n}{\left(x_{r,i}-x_{a}\right)}^{2}}$$ (20)

where $x_{a}$ represents the mean of all actual values.

## 4 Simulation verification

To verify the effectiveness and superiority of the proposed EMD-SSA-LSTM forecasting model, a comparative analysis was conducted against two benchmark models: a standalone LSTM model and an EMD-LSTM. These models were applied to the same time series dataset under identical experimental conditions. To ensure fairness and consistency in comparison, the following simulation parameters were carefully configured and kept uniform across all predictive schemes.

### 4.1 Dataset provenance and experimental setup

Device Under Test and Stress Conditions: The accelerated life test data was obtained from a population of commercial p-type MOSFETs with a gate oxide thickness of 2.5 nm. The devices were subjected to constant high-temperature gate bias (HTGB) stress, a standard accelerated test for invoking NBTI.

Data Acquisition: The $\Delta V_{th}$ was measured in-situ at predefined intervals using a precision semiconductor parameter analyzer (Keysight B1500A). The dataset comprises 512 time-series samples for each device, recorded at a fixed sampling interval of 1 hour until a predefined failure criterion of $\Delta V_{th}>50$ mV was reached. Data from multiple devices and stress conditions (T = 125^∘^C, 150^∘^C; Vgs = -2.0V, -2.2V) were aggregated to create a robust and generalizable training set.

Data Partitioning and Preprocessing: The aggregated dataset was partitioned chronologically on a per-device basis, with 70% of the sequence used for training, 15% for validation (for early stopping), and the final 15% held out for testing. This ensures the model is evaluated on unseen future degradation data, simulating a real-world prognostic scenario. All data was normalized to the [0, 1] range based on the training set’s minimum and maximum values to ensure stable and efficient network training.

### 4.2 Simulation parameter setting

The time series dataset employed in this study comprises voltage signal measurements with a total of 512 samples, collected at a fixed sampling interval of one hour. During the data preprocessing phase, the raw signal was restructured into input–output pairs suitable for supervised learning by applying a sliding window technique. The lookback window size was set to 5, meaning that each input sample consists of five consecutive historical voltage values. The forecast horizon was set to 1, indicating that the model predicts the voltage value at the next time step. This configuration effectively captures the temporal dependencies inherent in the time series, allowing the model to learn the dynamic influence of past states on future outcomes.

To better characterize the non-stationary nature of the voltage signal, EMD was applied to decompose the original signal into a set of IMFs. EMD adaptively separates a complex signal into a finite number of oscillatory components, each representing a distinct frequency band. In this study, the EMD procedure was configured with a bandwidth penalty parameter α=2500 to control the smoothness of the IMFs, and the DC component was suppressed by setting *DC* = 0. The frequency initialization method was set to *init* = 1, which uniformly distributes the initial center frequencies across the spectrum. A convergence tolerance of $tol=1\;\times \;10^{-7}$ was enforced to ensure decomposition accuracy. This setup enables effective extraction of signal features across multiple frequency scales, providing a refined basis for subsequent LSTM modeling.

An LSTM-based architecture served as the core forecasting model in this work. The network consists of an input layer, a single LSTM hidden layer, a ReLU activation layer, a fully connected regression layer, and a MSE loss function. The input dimension is determined by the lookback window size and the number of input features. For instance, in the case of a univariate voltage signal with a lookback window size of 5, the input dimension is 5. To ensure robust training and enhance generalization performance, the following hyperparameters were adopted: the maximum number of training epochs was set to 70, the initial learning rate to 0.01, and the gradient clipping threshold to 1. A learning rate schedule was applied, reducing the rate by a factor of 0.2 every 60 epochs. L2 regularization with a coefficient of 0.01 was incorporated to prevent overfitting, and the Adam optimizer was used for stochastic gradient descent. All experiments were executed on a computing node equipped with an Intel Xeon Gold 6248R processor (3.0 GHz base frequency) and 256 GB of RAM, utilizing the MATLAB R2021a programming environment. The average time to complete a full training cycle for the final EMD-SSA-LSTM model was approximately 529 seconds. Training progress was monitored in real-time via plotted loss curves. Prior to training, all input features were normalized to the range [0, 1] using min-max scaling to mitigate numerical instability and accelerate convergence.

### 4.3 Comparative analysis of forecasting performance

**Fig 5:**
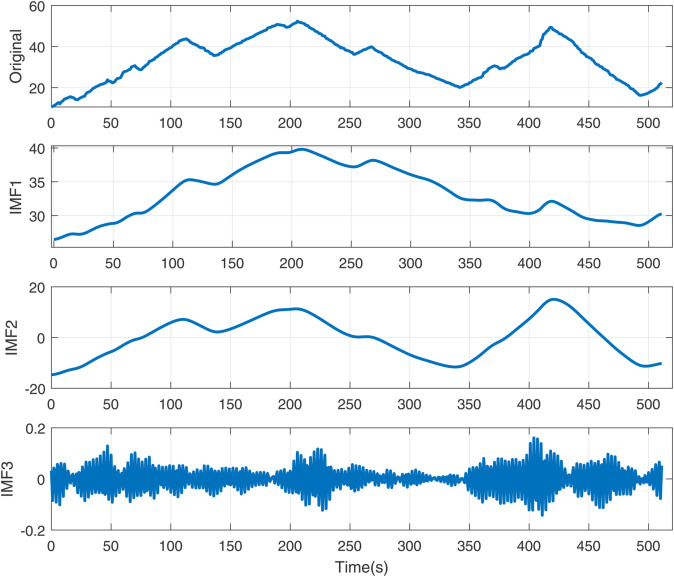
The decomposition diagram of EMD with modal number *K* = 3.

**Fig 6:**
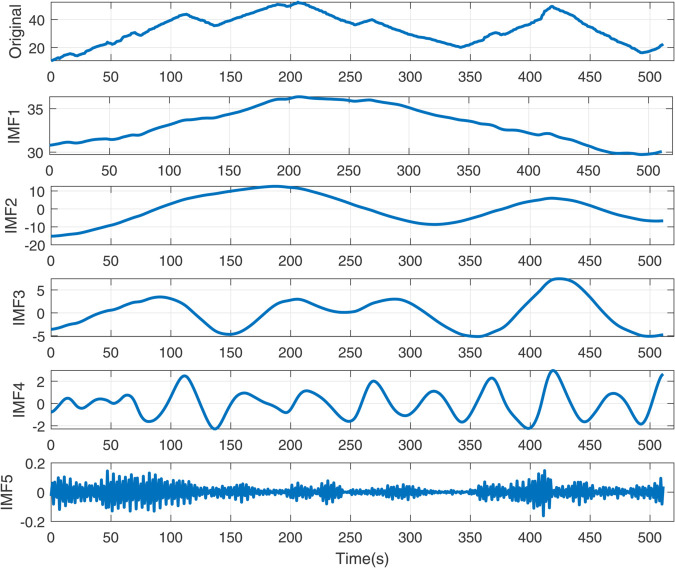
The decomposition diagram of EMD with modal number *K* = 5.

**Fig 7:**
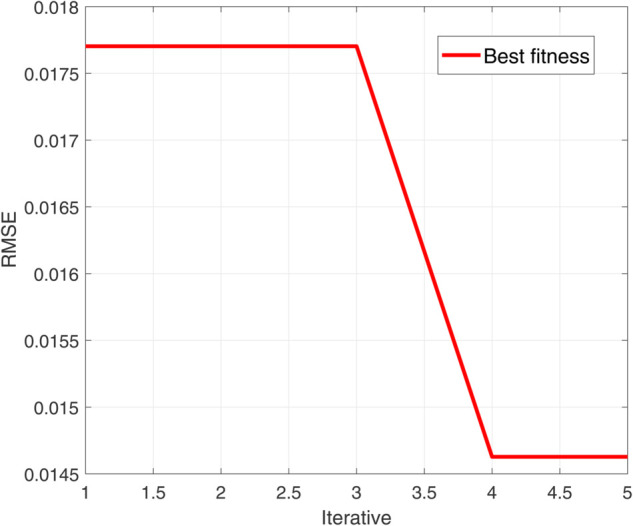
Evolution convergence diagram of SSA-LSTM prediction model.

**Fig 8:**
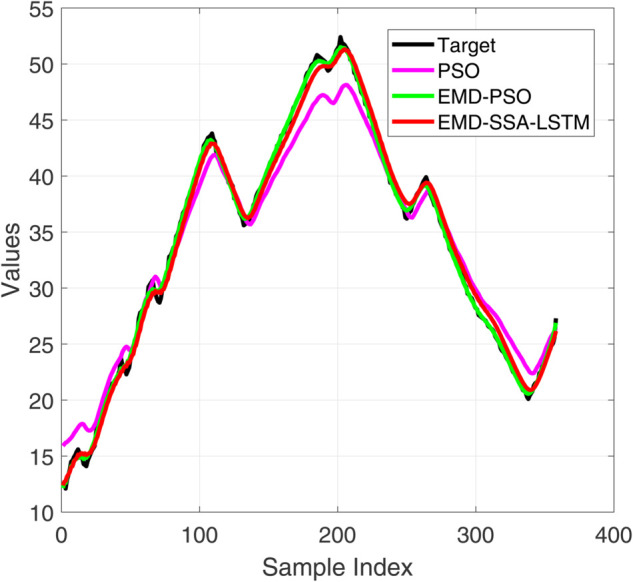
Comparative diagram of training set results of three different prediction models.

**Fig 9:**
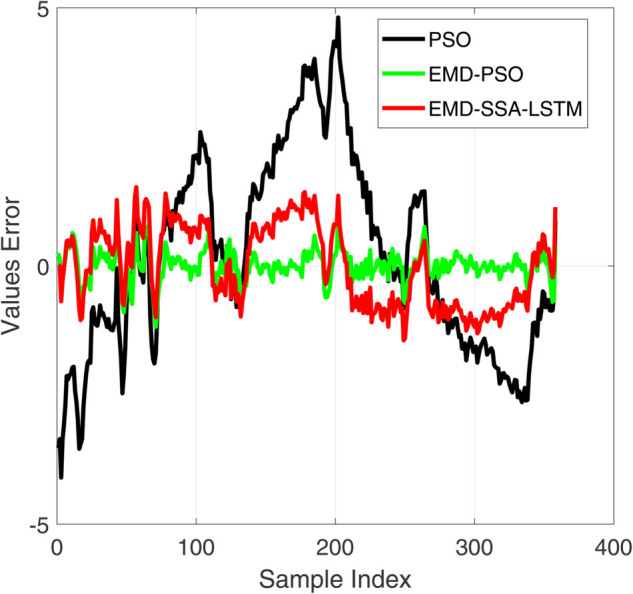
Comparative diagram of training set error results of three different prediction models.

**Fig 10:**
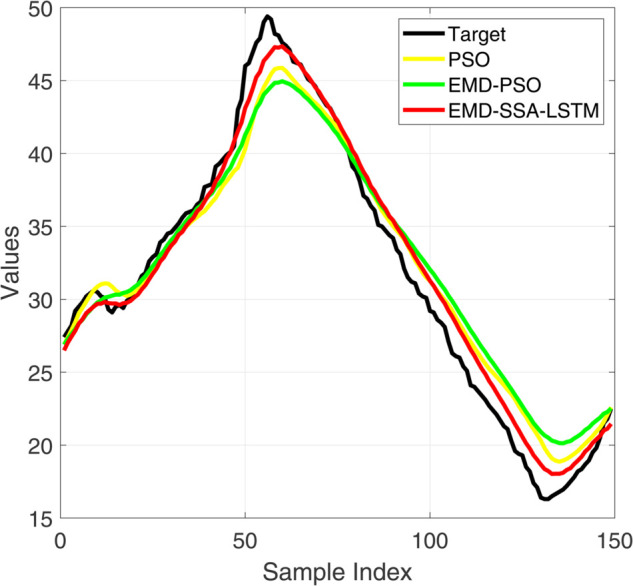
Comparative diagram of test set results of three different prediction models.

**Fig 11:**
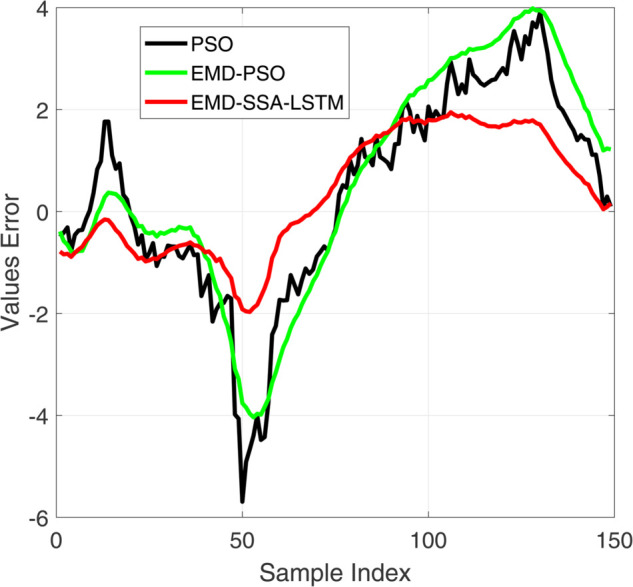
Comparative diagram of test set error results of three different prediction models.

The simulation results (Figs 5–11) systematically illustrate performance improvements from signal decomposition and hyperparameter optimization. Figs 5 and 6 compare decomposition granularity: with *K* = 3, the signal separates into trend, oscillation, and noise; with *K* = 5, finer quasi-periodic structures are isolated, improving feature separation at increased complexity. Fig 7 shows the SSA-LSTM optimization converging within five iterations. The RMSE remains near 0.0178 for the first three iterations, drops sharply to 0.0146 by the fourth, and stabilizes, confirming effective convergence.

Figs 8 and 10 show the performance of the training and testing sets of the prediction model. Visually, the trajectory predicted by our EMD-SSA-LSTM model (red line) is closer to the ground truth target (black line) on both the training and testing sets. In contrast, PSO-LSTM (blue) and EMD-PSO-LSTM (green) predictions show significant bias, particularly in capturing the exact amplitude and timing of voltage peaks. Figs 9 and 11 show the prediction errors of the prediction model. In terms of quantity, the prediction error curve of EMD-SSA-LSTM (red) has always been tighter and closer to zero, indicating lower bias and variance. The error band of the model based on particle swarm optimization is significantly wider and the peak amplitude is larger, especially on the test set, indicating poor generalization ability and stability. These new results strongly demonstrate that the SSA optimizer in our fusion architecture is more effective than mature PSO algorithms in finding high-performance LSTM configurations, resulting in higher prediction accuracy and generalization ability. This strengthens our claim for the novelty and practicality of SSA-LSTM fusion.

## 5 Discussion and future work

This study successfully establishes and validates an EMD-SSA-LSTM framework for predicting transistor threshold voltage drift ($\Delta V_{th}$). The proposed methodology, integrating EMD for signal denoising and feature extraction with SSA for automated LSTM hyperparameter optimization, demonstrates superior capability in capturing the complex, nonlinear dynamics of IC aging. Extensive validation under accelerated stress conditions confirms that our model achieves significantly higher accuracy and robustness compared to multiple benchmarks, including standalone LSTM, EMD-LSTM, and newly added PSO-optimized variants, providing an effective data-driven tool for high-precision IC reliability prognostics.

Future research will focus on three key directions: multi-objective SSA optimization to balance accuracy with computational efficiency; multi-sensor fusion architectures integrating additional degradation indicators like leakage current for enhanced robustness; and hybrid signal processing techniques combined with advanced neural architectures to improve adaptability across diverse IC technologies and operational profiles.

## Supporting information

S1 paper program (PDF)
