# Supplementary material for: Intelligence prediction of integrated circuit reliability based on SSA-LSTM fusion architecture
Source: PLoS One. 2025 Dec 31;20(12):e0339394. doi: 10.1371/journal.pone.0339394 (PMC12755746; doi:10.1371/journal.pone.0339394)
Supplement: S1 paper program — (PDF) [file pone.0339394.s001.pdf]

```
 tic

clc

clear all

fs=1;% Sampling frequency, i.e., the time interval between two data points in the time series, sampled at 1h intervals here

Ts=1/fs;% Sampling period


X = xlsread('voltage.xlsx','A1:A512');


save origin_data X


L=length(X);% Number of sampling points, i.e., how many data points there are

t=(0:L-1)*Ts;% Time series

STA=0; % Starting position of sampling, starting from hour 0 here


%--------- Some sample parameters for VMD: Setting parameters for VMD sample---------------

alpha = 2500;           % moderate bandwidth constraint

tau = 0;                % noise-tolerance (no strict fidelity enforcement)

K = 5;                  % number of modes

DC = 0;                 % no DC part imposed

init = 1;               % initialize omegas uniformly

tol = 1e-7

%--------------- Run actual VMD code: Perform VMD decomposition on data---------------------------

[u, u_hat, omega] = VMD(X(:,end), alpha, tau, K, DC, init, tol);


save vmd_data u
```

```
figure(1);

imfn=u;

n=size(imfn,1); % size(X,1) returns the number of rows of matrix X; size(X,2) returns the number of columns of matrix X; N=size(X,2) assigns the number of columns of matrix X to N

subplot(n+1,1,1);   % m represents rows, n represents columns, p represents which row and column this graph is plotted in. For example: subplot(2,2,[1,2])

plot(t,X(:,end));

ylabel('Original','fontsize',10);

% title('VMD Decomposition');


for n1=1:n

    subplot(n+1,1,n1+1);

    plot(t,u(n1,:));% Output IMF components, a(:,n) represents the elements of the nth column of matrix a, u(n1,:) represents the elements of the n1th row of matrix u

    ylabel(['IMF' int2str(n1)]);% int2str(i) converts numerical value i to a string after rounding, naming the y-axis

end

 xlabel('Time(s)','fontsize',12);


function [u, u_hat, omega] = VMD(signal, alpha, tau, K, DC, init, tol)

% Variational Mode Decomposition

% Authors: Konstantin Dragomiretskiy and Dominique Zosso

% zosso@math.ucla.edu --- http://www.math.ucla.edu/~zosso

% Initial release 2013-12-12 (c) 2013

%

% Input and Parameters:
```

```
% ---------------------
% signal    - the time domain signal (1D) to be decomposed
% alpha      - the balancing parameter of the data-fidelity constraint
% tau          - time-step of the dual ascent ( pick 0 for noise-slack )
% K             - the number of modes to be recovered
% DC           - true if the first mode is put and kept at DC (0-freq)
% init        - 0 = all omegas start at 0
%                                  1 = all omegas start uniformly distributed
%                                  2 = all omegas initialized randomly
% tol          - tolerance of convergence criterion; typically around 1e-6
%
% Output:
% -------
% u             - the collection of decomposed modes
% u_hat      - spectra of the modes
% omega      - estimated mode center-frequencies
%
% When using this code, please do cite our paper:
% -----------------------------------------------
% K. Dragomiretskiy, D. Zosso, Variational Mode Decomposition, IEEE Trans.
% on Signal Processing (in press)
% please check here for update reference:
%                   http://dx.doi.org/10.1109/TSP.2013.2288675
%---------- Preparations


% Period and sampling frequency of input signal
save_T = length(signal);
```

```
fs = 1/save_T;

% extend the signal by mirroring
T = save_T;
f_mirror(1:T/2) = signal(T/2:-1:1);
f_mirror(T/2+1:3*T/2) = signal;
f_mirror(3*T/2+1:2*T) = signal(T:-1:T/2+1);
f = f_mirror;

% Time Domain 0 to T (of mirrored signal)
T = length(f);
t = (1:T)/T;

% Spectral Domain discretization
freqs = t-0.5-1/T;

% Maximum number of iterations (if not converged yet, then it won't anyway)
N = 500;

% For future generalizations: individual alpha for each mode
Alpha = alpha*ones(1,K);

% Construct and center f_hat
f_hat = fftshift((fft(f)));
f_hat_plus = f_hat;
f_hat_plus(1:T/2) = 0;
```

```
% matrix keeping track of every iterant // could be discarded for mem
u_hat_plus = zeros(N, length(freqs), K);

% Initialization of omega_k
omega_plus = zeros(N, K);
switch init
    case 1
        for i = 1:K
            omega_plus(1,i) = (0.5/K)*(i-1);
        end
    case 2
        omega_plus(1,:) = sort(exp(log(fs) + (log(0.5)-log(fs))*rand(1,K)));
    otherwise
        omega_plus(1,:) = 0;
end

% if DC mode imposed, set its omega to 0
if DC
    omega_plus(1,1) = 0;
end

% start with empty dual variables
lambda_hat = zeros(N, length(freqs));

% other inits
uDiff = tol+eps; % update step
n = 1; % loop counter
```

```
sum_uk = 0; % accumulator

% ----------- Main loop for iterative updates
while ( uDiff > tol &&   n < N ) % not converged and below iterations limit

    % update first mode accumulator
    k = 1;
    sum_uk = u_hat_plus(n,:,K) + sum_uk - u_hat_plus(n,:,1);

    % update spectrum of first mode through Wiener filter of residuals
    u_hat_plus(n+1,:,k) = (f_hat_plus - sum_uk - 
lambda_hat(n,:)/2)./(1+Alpha(1,k)*(freqs - omega_plus(n,k)).^2);

    % update first omega if not held at 0
    if ~DC
        omega_plus(n+1,k) = (freqs(T/2+1:T)*(abs(u_hat_plus(n+1, T/2+1:T, 
k)).^2)')/sum(abs(u_hat_plus(n+1,T/2+1:T,k)).^2);
    end

    % update of any other mode
    for k=2:K

        % accumulator
        sum_uk = u_hat_plus(n+1,:,k-1) + sum_uk - u_hat_plus(n,:,k);

        % mode spectrum
        u_hat_plus(n+1,:,k) = (f_hat_plus - sum_uk - 
lambda_hat(n,:)/2)./(1+Alpha(1,k)*(freqs - omega_plus(n,k)).^2);
```

```
            % center frequencies
            omega_plus(n+1,k) = (freqs(T/2+1:T)*(abs(u_hat_plus(n+1, T/2+1:T,
k)).^2)')/sum(abs(u_hat_plus(n+1,T/2+1:T,k)).^2);

    end

    % Dual ascent
    lambda_hat(n+1,:) = lambda_hat(n,:) + tau*(sum(u_hat_plus(n+1,:,:),3) - f_hat_plus);

    % loop counter
    n = n+1;

    % converged yet?
    uDiff = eps;
    for i=1:K
        uDiff = uDiff + 1/T*(u_hat_plus(n,:,i)-u_hat_plus(n-1,:,i))*conj((u_hat_plus(n,:,i)-
u_hat_plus(n-1,:,i)))';
    end
    uDiff = abs(uDiff);

end
%------ Postprocessing and cleanup
% discard empty space if converged early
N = min(N,n);
omega = omega_plus(1:N,:);
```

```
% Signal reconstruction
u_hat = zeros(T, K);
u_hat((T/2+1):T,:) = squeeze(u_hat_plus(N,(T/2+1):T,:));
u_hat((T/2+1):-1:2,:) = squeeze(conj(u_hat_plus(N,(T/2+1):T,:)));
u_hat(1,:) = conj(u_hat(end,:));

u = zeros(K,length(t));

for k = 1:K
    u(k,:)=real(ifft(ifftshift(u_hat(:,k))));
end

% remove mirror part
u = u(:,T/4+1:3*T/4);

% recompute spectrum
clear u_hat;
for k = 1:K
    u_hat(:,k)=fftshift(fft(u(k,:)))';
end
end

%______________________________________________________________________%
% Sparrow Search Algorithm (SSA) %
%______________________________________________________________________%
function [Best_pos,Best_score,curve,BestNet]=SSA(pop,Max_iter,lb,ub,dim,fobj)
disp('Sparrow Search Algorithm starting...')
```

```
ST = 0.6;% Safety threshold
PD = 0.7;% Proportion of producers, the rest are scroungers
SD = 0.2;% Proportion of sparrows who sense danger

PDNumber = round(pop*PD); % Number of producers
SDNumber = round(pop*SD);% Number of sparrows who sense danger
if(max(size(ub)) == 1)
    ub = ub.*ones(1,dim);
    lb = lb.*ones(1,dim);
end
net = {};
% Population initialization
X0=initialization(pop,dim,ub,lb);
X = X0;
% Calculate initial fitness values
fitness = zeros(1,pop);
for i = 1:pop
    [fitness(i),net{i}] =   fobj(X(i,:));
end
  [fitness, index]= sort(fitness);% Sorting
BestF = fitness(1);
WorstF = fitness(end);
GBestF = fitness(1);% Global best fitness value
for i = 1:pop
     X(i,:) = X0(index(i),:);
     net{i}=net{index(i)};
end
```

```
curve=zeros(1,Max_iter);
GBestX = X(1,:);% Global best position
X_new = X;
BestNet = net{1};
curve(1)=GBestF;
for i = 2: Max_iter
    disp(['Iteration ',num2str(i)]);
    BestF = fitness(1);
    WorstF = fitness(end);


    R2 = rand(1);
   for j = 1:PDNumber
      if(R2<ST)
          X_new(j,:) = X(j,:).*exp(-j/(rand(1)*Max_iter));
      else
          X_new(j,:) = X(j,:) + randn()*ones(1,dim);
      end
   end
   for j = PDNumber+1:pop
%          if(j>(pop/2))
         if(j>(pop - PDNumber)/2 + PDNumber)
          X_new(j,:)= randn().*exp((X(end,:) - X(j,:))/j^2);
       else
          % Generate random numbers between -1 and 1
          A = ones(1,dim);
          for a = 1:dim
```

```
                    if(rand()>0.5)
                        A(a) = -1;
                    end
                end
                AA = A'*inv(A*A');
                X_new(j,:)= X(1,:) + abs(X(j,:) - X(1,:)).*AA';
            end
        end
        Temp = randperm(pop);
        SDchooseIndex = Temp(1:SDNumber);
        for j = 1:SDNumber
            if(fitness(SDchooseIndex(j))>BestF)
                X_new(SDchooseIndex(j),:) = X(1,:) + randn().*abs(X(SDchooseIndex(j),:) -
X(1,:));
            elseif(fitness(SDchooseIndex(j))== BestF)
                K = 2*rand() -1;
                X_new(SDchooseIndex(j),:) = X(SDchooseIndex(j),:) +
K.*(abs( X(SDchooseIndex(j),:) - X(end,:))./(fitness(SDchooseIndex(j)) - fitness(end) +
10^-8));
            end
        end
        % Boundary control
        for j = 1:pop
            for a = 1: dim
                if(X_new(j,a)>ub(a)||isnan(X_new(j,a)))% isnan() function determines if a
value is NaN, if NaN then take upper bound
                    X_new(j,a) =ub(a);
                end
```

```
                if(X_new(j,a)<lb(a)||isnan(X_new(j,a)))
                        X_new(j,a) =lb(a);
                end
        end
end
% Update position
for j=1:pop
  [fitness_new(j),net{j}] = fobj(X_new(j,:));
end
for j = 1:pop
  if(fitness_new(j) < GBestF)
        GBestF = fitness_new(j);
          GBestX = X_new(j,:);
          BestNet=net{j};
  end
end
X = X_new;
fitness = fitness_new;
  % Sorting update
[fitness, index]= sort(fitness);% Sorting
BestF = fitness(1);
WorstF = fitness(end);
for j = 1:pop
      X(j,:) = X(index(j),:);
      net{j}=net{index(j)};
end
curve(i) = GBestF;
```

```
end

Best_pos =GBestX;

Best_score = curve(end);

End


clc;

clear

close all


%% LSTM Prediction

tic

load origin_data.mat

load vmd_data.mat


disp('…………………………………………………………………………')

disp('Single LSTM Prediction')

disp('…………………………………………………………………………')


num_samples = length(X);             % Number of samples

kim = 5;                             % Time delay steps (using kim historical data as
independent variables)

zim =   1;                           % Predict across zim time points

or_dim = size(X,2);


%   Reconstruct dataset

for i = 1: num_samples - kim - zim + 1

    res(i, :) = [reshape(X(i: i + kim - 1,:), 1, kim*or_dim), X(i + kim + zim - 1,:)];
```

```
end

% Training set and test set division
outdim = 1;                                           % Last column as output
num_size = 0.7;                                       % Proportion of training set in dataset
num_train_s = round(num_size * num_samples); % Number of training samples
f_ = size(res, 2) - outdim;                           % Input feature dimension

P_train = res(1: num_train_s, 1: f_)';
T_train = res(1: num_train_s, f_ + 1: end)';
M = size(P_train, 2);

P_test = res(num_train_s + 1: end, 1: f_)';
T_test = res(num_train_s + 1: end, f_ + 1: end)';
N = size(P_test, 2);

%   Data normalization
[p_train, ps_input] = mapminmax(P_train, 0, 1);
p_test = mapminmax('apply', P_test, ps_input);

[t_train, ps_output] = mapminmax(T_train, 0, 1);
t_test = mapminmax('apply', T_test, ps_output);

%   Format conversion
for i = 1 : M
```

```
    vp_train{i, 1} = p_train(:, i);
    % After reconstruction: 24 inputs, 1 output, total 1571 rows
    % Through this format conversion:
    % When i=1, pack the 24 inputs of the first row into a 1x1 cell
    % Similarly, becomes 1571 cells arranged in a column
    % The 24 numbers inside one cell are arranged vertically as 24x1
    % Cell data format is double
    vt_train{i, 1} = t_train(:, i);
end

for i = 1 : N
    vp_test{i, 1} = p_test(:, i);
    vt_test{i, 1} = t_test(:, i);
end

%   Create LSTM network
layers = [ ...
    sequenceInputLayer(f_)              % Input layer
    lstmLayer(70)                       % LSTM layer
    reluLayer                           % Relu activation layer
    fullyConnectedLayer(outdim)         % Regression layer
    regressionLayer];

%   Parameter settings
options = trainingOptions('adam', ...             % Optimizer: Adam
    'MaxEpochs', 70, ...                          % Maximum training epochs
    'GradientThreshold', 1, ...                   % Gradient threshold
```

```
        'InitialLearnRate', 0.01, ...                  % Initial learning rate
        'LearnRateSchedule', 'piecewise', ...                      % Learning rate schedule
        'LearnRateDropPeriod', 60, ...                              % Start adjusting learning rate
after 850 iterations
        'LearnRateDropFactor',0.2, ...                              % Learning rate drop factor
        'L2Regularization', 0.01, ...                % Regularization parameter
        'ExecutionEnvironment', 'cpu',...                           % Training environment
        'Verbose', 0, ...                                              % Turn off optimization
process
        'Plots', 'training-progress');                              % Plot training progress

%   Training
net = trainNetwork(vp_train, vt_train, layers, options);
%analyzeNetwork(net);% View network structure
%   Prediction
t_sim1 = predict(net, vp_train);
t_sim2 = predict(net, vp_test);

%   Data denormalization
T_sim1 = mapminmax('reverse', t_sim1, ps_output);
T_sim2 = mapminmax('reverse', t_sim2, ps_output);
T_train1 = T_train;
T_test2 = T_test;

%   Data format conversion
T_sim1 = cell2mat(T_sim1);% cell2mat converts cell array to regular array
T_sim2 = cell2mat(T_sim2);
```

```
% Calculate evaluation metrics
disp('Training set error metrics')
[mae1,rmse1,mape1,error1]=calc_error(T_train1,T_sim1');
fprintf('\n')

disp('Test set error metrics')
[mae2,rmse2,mape2,error2]=calc_error(T_test2,T_sim2');
fprintf('\n')
toc


tic
disp('…………………………………………………………………………')
disp('VMD-LSTM Prediction')
disp('…………………………………………………………………………')

imf=u;
c=size(imf,1);
%% Modeling each component
for d=1:c
disp(['Modeling component ',num2str(d)])

X_imf=[X(:,1:end-1) imf(d,:)'];
num_samples = length(X_imf);    % Number of samples

%    Reconstruct dataset
```

```
for i = 1: num_samples - kim - zim + 1
    res(i, :) = [reshape(X_imf(i: i + kim - 1,:), 1, kim*or_dim), X_imf(i + kim + zim - 1,:)];
end


% Training set and test set division
outdim = 1;                                   % Last column as output
num_size = 0.7;                               % Proportion of training set in dataset
num_train_s = round(num_size * num_samples); % Number of training samples
f_ = size(res, 2) - outdim;                   % Input feature dimension


P_train = res(1: num_train_s, 1: f_)';
T_train = res(1: num_train_s, f_ + 1: end)';


P_test = res(num_train_s + 1: end, 1: f_)';
T_test = res(num_train_s + 1: end, f_ + 1: end)';


%  Data normalization
[p_train, ps_input] = mapminmax(P_train, 0, 1);
p_test = mapminmax('apply', P_test, ps_input);

[t_train, ps_output] = mapminmax(T_train, 0, 1);
t_test = mapminmax('apply', T_test, ps_output);
```

```
%   Format conversion
for i = 1 : M
    vp_train{i, 1} = p_train(:, i);
    vt_train{i, 1} = t_train(:, i);
end

for i = 1 : N
    vp_test{i, 1} = p_test(:, i);
    vt_test{i, 1} = t_test(:, i);
end

%   Create LSTM network
layers = [ ...
    sequenceInputLayer(f_)                  % Input layer
    lstmLayer(70)                           % LSTM layer
    reluLayer                               % Relu activation layer
    fullyConnectedLayer(outdim)             % Regression layer
    regressionLayer];

%   Parameter settings
options = trainingOptions('adam', ...                   % Optimizer: Adam
    'MaxEpochs', 70, ...                                % Maximum training epochs
    'GradientThreshold', 1, ...                         % Gradient threshold
    'InitialLearnRate', 0.01, ...           % Initial learning rate
    'LearnRateSchedule', 'piecewise', ...               % Learning rate schedule
    'LearnRateDropPeriod', 60, ...                      % Start adjusting learning rate
after 850 iterations
```

```
        'LearnRateDropFactor',0.2, ...                              % Learning rate drop factor
        'L2Regularization', 0.01, ...                % Regularization parameter
        'ExecutionEnvironment', 'cpu',...                           % Training environment
        'Verbose', 0, ...                                             % Turn off optimization
process
        'Plots', 'training-progress');                              % Plot training progress

%   Training
net = trainNetwork(vp_train, vt_train, layers, options);
%   Prediction
t_sim5 = predict(net, vp_train);
t_sim6 = predict(net, vp_test);

%   Data denormalization
T_sim5_imf = mapminmax('reverse', t_sim5, ps_output);
T_sim6_imf = mapminmax('reverse', t_sim6, ps_output);

%   Data format conversion
T_sim5(d,:) = cell2mat(T_sim5_imf);% cell2mat converts cell array to regular array
T_sim6(d,:) = cell2mat(T_sim6_imf);
T_train5(d,:)= T_train;
T_test6(d,:)= T_test;
end

% Sum the prediction results of each component
T_sim5=sum(T_sim5);
T_sim6=sum(T_sim6);
```

```
T_train5=sum(T_train5);
T_test6=sum(T_test6);

% Calculate evaluation metrics
disp('Training set error metrics')
[mae5,rmse5,mape5,error5]=calc_error(T_train5,T_sim5);
fprintf('\n')

disp('Test set error metrics')
[mae6,rmse6,mape6,error6]=calc_error(T_test6,T_sim6);
fprintf('\n')
toc

%% VMD-SSA-LSTM Prediction
tic
disp('…………………………………………………………………………')
disp('VMD-SSA-LSTM Prediction')
disp('…………………………………………………………………………')

% SSA parameter settings
pop=3; % Population size
Max_iter=5; % Maximum number of iterations
dim=3; % Optimize 3 parameters of LSTM
lb = [50,50,0.001];% Lower bounds
ub = [300,300,0.01];% Upper bounds
numFeatures=f_;
numResponses=outdim;
```

```
fobj = @(x) fun(x,numFeatures,numResponses,X) ;
[Best_pos,Best_score,curve,BestNet]=SSA(pop,Max_iter,lb,ub,dim,fobj);

% Plot evolution curve
figure
plot(curve,'r-','linewidth',3)
xlabel('Evolution Generation')
ylabel('Root Mean Square Error RMSE')
legend('Best Fitness')
title('Evolution Convergence Curve of SSA-LSTM')

disp('')
disp(['Optimal number of hidden units:      ',num2str(round(Best_pos(1)))]);
disp(['Optimal maximum training epochs:      ',num2str(round(Best_pos(2)))]);
disp(['Optimal initial learning rate:      ',num2str((Best_pos(3)))]);

%% Modeling each component
for d=1:c
disp(['Modeling component ',num2str(d)])

X_imf=[X(:,1:end-1) imf(d,:)'];

%    Reconstruct dataset
for i = 1: num_samples - kim - zim + 1
    res(i, :) = [reshape(X_imf(i: i + kim - 1,:), 1, kim*or_dim), X_imf(i + kim + zim - 1,:)];
end
```

```
% Training set and test set division
outdim = 1;                                                       % Last column as output
num_size = 0.7;                                                   % Proportion of training set in dataset
num_train_s = round(num_size * num_samples); % Number of training samples
f_ = size(res, 2) - outdim;                                % Input feature dimension


P_train = res(1: num_train_s, 1: f_)';
T_train = res(1: num_train_s, f_ + 1: end)';
M = size(P_train, 2);

P_test = res(num_train_s + 1: end, 1: f_)';
T_test = res(num_train_s + 1: end, f_ + 1: end)';
N = size(P_test, 2);

%   Data normalization
[p_train, ps_input] = mapminmax(P_train, 0, 1);
p_test = mapminmax('apply', P_test, ps_input);

[t_train, ps_output] = mapminmax(T_train, 0, 1);
t_test = mapminmax('apply', T_test, ps_output);

%   Format conversion
for i = 1 : M
    vp_train{i, 1} = p_train(:, i);
    vt_train{i, 1} = t_train(:, i);
```

```
end

for i = 1 : N
        vp_test{i, 1} = p_test(:, i);
        vt_test{i, 1} = t_test(:, i);
end

% LSTM prediction with optimal parameters
layers = [ ...
        sequenceInputLayer(f_)                    % Input layer
        lstmLayer(round(Best_pos(1)))          % LSTM layer
        reluLayer                                  % Relu activation layer
        fullyConnectedLayer(outdim)               % Regression layer
        regressionLayer];



options = trainingOptions('adam', ...                          % Optimizer: Adam
        'MaxEpochs', round(Best_pos(2)), ...                   % Maximum training epochs
        'GradientThreshold', 1, ...                             % Gradient threshold
        'InitialLearnRate', Best_pos(3), ...                  % Initial learning rate
        'LearnRateSchedule', 'piecewise', ...                  % Learning rate schedule
        'LearnRateDropPeriod', round(Best_pos(2)*0.9), ... % Start adjusting learning rate
after training
        'LearnRateDropFactor',0.2, ...                          % Learning rate drop factor
        'L2Regularization', 0.001, ...                         % Regularization parameter
        'ExecutionEnvironment', 'cpu',...                      % Training environment
        'Verbose', 0, ...                                        % Turn off optimization
```

```
process
    'Plots', 'training-progress');                    % Plot training progress

%   Training
net = trainNetwork(vp_train, vt_train, layers, options);
%   Prediction
t_sim7 = predict(net, vp_train);
t_sim8 = predict(net, vp_test);

%   Data denormalization
T_sim7_imf = mapminmax('reverse', t_sim7, ps_output);
T_sim8_imf = mapminmax('reverse', t_sim8, ps_output);

%   Data format conversion
T_sim7(d,:) = cell2mat(T_sim7_imf);% cell2mat converts cell array to regular array
T_sim8(d,:) = cell2mat(T_sim8_imf);
T_train7(d,:)= T_train;
T_test8(d,:)= T_test;
end

% Sum the prediction results of each component
T_sim7=sum(T_sim7);
T_sim8=sum(T_sim8);
T_train7=sum(T_train7);
T_test8=sum(T_test8);

% Calculate evaluation metrics
```

```
disp('Training set error metrics')
[mae7,rmse7,mape7,error7]=calc_error(T_train7,T_sim7);
fprintf('\n')

disp('Test set error metrics')
[mae8,rmse8,mape8,error8]=calc_error(T_test8,T_sim8);
fprintf('\n')
toc

%% Comparison of four models - training set results plotting

figure
plot(T_train1,'k','linewidth',3);
hold on;
plot(T_sim1,'m','linewidth',3);
hold on;
plot(T_train5,'g','linewidth',3);
hold on;
plot(T_sim7,'r','linewidth',3);
legend('Target','PSO','EMD-PSO','EMD-SSA-LSTM');
%title('Comparative diagram of training set results of three prediction models');
xlabel('Sample Index');
ylabel('Values');
grid on;


figure
```

```
plot(T_train1-T_sim1','k','linewidth',3);
hold on
plot(T_train1-T_train5,'g','linewidth',3);
hold on
plot(T_train1-T_sim7,'r','linewidth',3);
legend('PSO','EMD-PSO','EMD-SSA-LSTM');
%title('Comparative diagram of training set error results of three prediction models');
grid on;



%%%%%%%%%%%%%%%%%%%%%%%%%%%%%%%%%%%%%%


%% Comparison of four models - test set results plotting


figure
plot(T_test2,'k','linewidth',3);
hold on;
plot(T_sim2,'y','linewidth',3);
hold on;
plot(T_sim6,'g','linewidth',3);
hold on;
plot(T_sim8,'r','linewidth',3);
legend('Target','PSO','EMD-PSO','EMD-SSA-LSTM');
%title('Comparative diagram of test set results of three prediction models');
xlabel('Sample Index');
ylabel('Values');
grid on;
```

```
figure
plot(error2,'k','linewidth',3);
hold on
plot(error6,'g','linewidth',3);
hold on
plot(error8,'r','linewidth',3);
legend('PSO','EMD-PSO','EMD-SSA-LSTM');
%title('Comparative diagram of test set results of three prediction models');
grid on;
```
